# Gender and anxiety reveal distinct computational sources of underconfidence

**DOI:** 10.1017/S0033291725102808

**Published:** 2026-01-15

**Authors:** Sucharit Katyal, Stephen M. Fleming

**Affiliations:** 1 Department of Psychology, University of Copenhagen, Denmark; 2 Max Planck UCL Centre for Computational Psychiatry and Ageing Research, University College London, UK; 3 Department of Experimental Psychology and Institute of Cognitive Neuroscience, University College London, UK

**Keywords:** anxiety, computational modelling, computational psychiatry, drift-diffusion models, evidence accumulation models, gender, metacognitive bias, metacognition, underconfidence

## Abstract

**Background:**

Confidence exhibits systematic individual differences across mental health, gender, and age. However, it remains unknown whether these distinct sources of metacognitive bias have common or distinct computational origins.

**Methods:**

To address this question, we developed a novel dynamic computational model of metacognition to study the temporal evolution of underconfidence associated with individual differences in transdiagnostic anxiety symptoms and gender in samples of online participants (total *N* = 1,447).

**Results:**

We found that underconfidence associated with anxiety symptoms became more prominent the longer individuals took to make metacognitive judgments – suggesting that it is exacerbated by additional time for introspection. In contrast, gender-related underconfidence decreased with greater metacognitive judgment time – suggesting that additional time for introspection is able to remediate prepotent biases. Our computational model of confidence explained these effects – while both gender and anxiety symptoms involved shifts in confidence criteria, only anxiety symptoms involved a temporal accumulation of negatively biased evidence about one’s ability.

**Conclusions:**

Our study reveals multiple computational pathways to the formation of underconfidence, in turn highlighting specific potential mechanisms for its remediation.

## Introduction

Humans possess a metacognitive capacity to reflect on their behavior and cognitive abilities in a variety of domains (Bandura, 1977; Flavell, 1979; Katyal & Fleming, 2024). In the laboratory, metacognition can be quantified by asking participants to provide confidence estimates in their task performance. A variety of individual differences predict features of confidence and metacognition, over and above their influence on task performance. For instance, transdiagnostic symptoms of anxiety and depression predict underconfidence, whereas transdiagnostic symptoms of compulsivity predict overconfidence (Benwell et al., 2022; Hoven et al., 2023; Katyal, Huys, Dolan, & Fleming, 2025; Rouault, Seow, Gillan, & Fleming, 2018; Seow & Gillan, 2020). Confidence is also influenced by gender, with women typically exhibiting underconfidence compared to men (Cooke-Simpson & Voyer, 2007; Hoogervorst et al., 2024; Jakobsson, 2012; Lundeberg, Fox, & Punćcohaŕ, 1994; Ross, Scott, & Bruce, 2012; although see Xue et al., 2024). However, it remains unknown whether these distinct sources of underconfidence have common or distinct computational origins. In other words, are anxious people underconfident for the same reasons as women are underconfident? Here, we investigated this question by asking how distinct individual differences influence the temporal dynamics of confidence formation.

**Figure 1.**
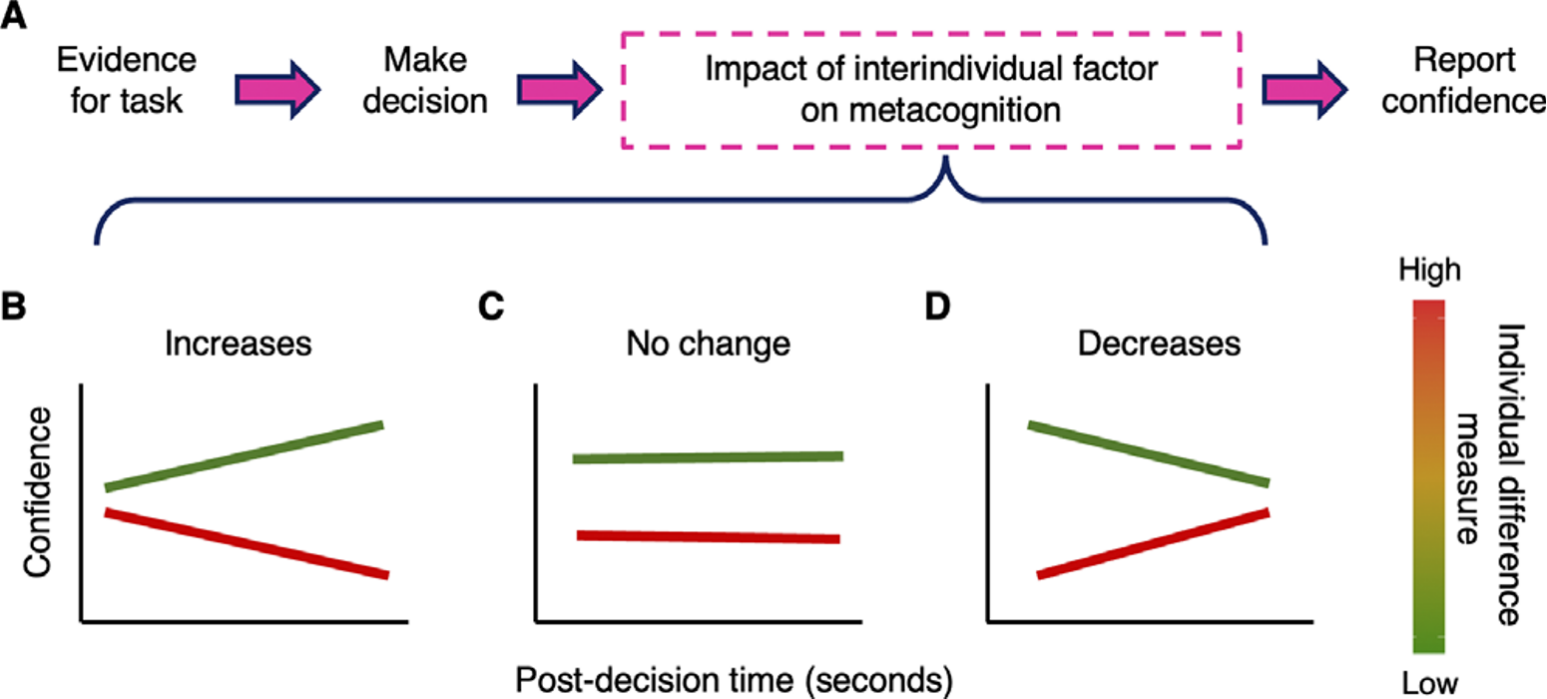
(a) Confidence is distorted (toward over- or underconfidence) by several individual difference factors. We studied how these distortions unfold over time in the period after making a decision, but before reporting one’s confidence. Consider an individual difference factor related to underconfidence (e.g. Anxiety) where a higher score on the factor (i.e. red on the color bar) is related to lower confidence and a lower score (green) to higher confidence. This distortion is depicted in panels (b)–(d) as lower confidence values for higher individual scores and vice versa. With post-decision time, such distortions in confidence can either (b) increase following a decision, (c) stay the same, or (d) decrease over time.

Recent work has shown that post-decisional processing makes a substantial contribution to the formation of metacognitive judgments, consistent with an active process of introspection on immediately preceding task performance. Specifically, confidence better matches performance if confidence reports are given after a delay rather than immediately following a decision (Desender, Vermeylen, & Verguts, 2022; Hellmann, Zehetleitner, & Rausch, 2023; Pleskac & Busemeyer, 2010). This indicates a speed-accuracy trade-off in confidence formation where metacognitive efficiency (i.e. the accuracy of confidence estimates) improves with the time taken to provide a metacognitive judgment (Desender et al., 2022). A natural question then arises as to how and whether individual differences in metacognitive biases, such as underconfidence, are expressed during this post-decisional period. One possibility is that underconfidence is exacerbated with increased time for post-decisional reflection – perhaps allowing more global factors (such as those arising from states of anxiety, or other prior beliefs; Van Marcke, Denmat, Verguts, & Desender, 2024) to contaminate the formation of confidence. In contrast, metacognitive biases may be attenuated with greater time for post-decisional reflection – perhaps allowing prepotent responses (such as a prior assumption that I am not confident) to be overridden with evidence accumulated from performance. These alternative possibilities are displayed graphically in Figure 1.

In the current study, we sought to distinguish between these different computational hypotheses by investigating how the expression of underconfidence is modulated by post-decisional processing. We availed ourselves of published datasets in which underconfidence has been documented in relation to Anxiety symptoms (Katyal et al., 2025; Rouault et al., 2018), and where data on the Gender of participants and post-decisional (confidence) response times were also available. We asked whether the influence of Anxiety symptoms and Gender on confidence interacted with confidence response times. We also asked whether different computational features of post-decisional processing, including evidence accumulation and the setting of confidence criteria, could account for the pattern of our findings. To pre-empt our results, we found that while underconfidence associated with Anxiety symptoms *increased* in individuals who took more time for post-decisional reflection, underconfidence associated with Gender instead *decreased* with post-decision time. Computational modeling revealed that these differences could be explained by a post-decisional accumulation of negatively biased evidence about performance that was specific to mental ill health.

## Methods

### Participants and exclusions

We identified four published large datasets from two studies (Katyal et al., 2025; Rouault et al., 2018) that measured trial-by-trial confidence ratings in two-alternative forced choice (2-AFC) tasks along with individual difference measures of Anxiety-Depression and Gender, in addition to confidence rating response times. The datasets also contained information on participants’ age, and two of the datasets also measured transdiagnostic symptoms of compulsivity; in the Supplementary Material, we report exploratory analyses of these individual differences. The datasets had a combined *N* = 1,647 (see Supplementary Table 1 for sample sizes of individual studies).

We excluded trials where choice and confidence response times were >3 median absolute deviations from the median within each task within a dataset. For Experiments 3 and 4, participants provided confidence ratings on discrete 11- and 5-point scales. We excluded participants whose variability in confidence ratings, measured as standard deviation across all confidence reports, was less than the resolution of the discrete rating scale (for a 5-point scale, the resolution would be ⅕). After exclusions, *N* = 1,447 participants remained for analysis.

### Tasks

Multiple 2-AFC tasks were used in different datasets, details of which can be found in the original articles (Katyal et al., 2025; Rouault et al., 2018). Briefly, Experiments 1 and 2 employed two gamified tasks – a perceptual discrimination task where participants chose which of two colors of stimuli were presented in larger numbers, and a visual working memory task requiring memorizing several objects for a short period and choosing which of two stimuli was present in the array (with one of the options being a novel stimulus). After making their choice on the task, participants were asked to report confidence in their choice by selecting a continuous value between 0 and 100% on a vertical slider. Here, confidence reaction times (RTs) were measured as the time between choice and the time they made their confidence selection. On most trials, participants selected their confidence with one click, making it straightforward to define confidence RTs. On trials in which participants dragged the slider, confidence RT was defined as the time at which the slider arrived at its final position. Experiments 3 and 4 used a perceptual task where participants were shown two stimulus arrays containing several dots on the left and right side and asked to discriminate which array contained a larger number of dots. Participants reported their choice, followed by a confidence report. To report confidence, participants clicked on a horizontal slider with discrete intervals and clicked a submit button below the slider to indicate their selected confidence. Confidence RTs were measured as the time between the choice and the time the submit button was pressed. Choice RTs < 100 ms, and choice and confidence RTs > 3 median absolute deviations from the median (calculated for each task for each experiment) were excluded from analyses.

### Individual difference measures

Experiments 1 and 3 used the Generalized Anxiety Disorder 7-item questionnaire to measure general anxiety, which we used as the Anxiety score. For Experiments 2 and 4, Anxiety (and Compulsivity) scores were measured transdiagnostically (Gillan et al., 2016; Hopkins et al., 2022). For combining across datasets, Anxiety and Compulsivity scores were *z*-scored within each dataset.

For Experiments 1 and 2, Gender was measured through one of three self-report options: man, woman, and nonbinary. Three participants who selected nonbinary were excluded from the analyses of Gender individual differences.

### Statistical analysis

Statistical analyses (and figure generation) were done in R (v 4.3.1). Mixed-effects regression was performed using the *lmer* and *glmer* functions from the library *lmerTest* (v 3.1). Marginals for the interaction effects were plotted using the *plot_model* function from the library *sjPlot* (v 2.8.14).

For model-free analyses, we performed linear regressions after *z*-scoring individual difference factors (except Gender and Age), RTs, confidence ratings, and task difficulty within datasets (separately for the two tasks in Experiments 1 and 2) to allow pooling across datasets and tasks. *Z*-scored confidence ratings, RTs, and task difficulty were then averaged across participants.

To test how different individual difference factors were associated with confidence, we constructed the following regression model:

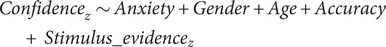

Here, 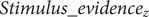 is the within-task and within-experiment *z*-scored value of the behavioral staircase (higher values indicate lower difficulty). This value was defined as the difference in numbers of the two types of stimuli per trial for the perception task in Experiments 1 and 2, the set size on a trial subtracted from the maximum possible set size for the memory task in Experiments 1 and 2, and the difference in dots per trial for Experiments 3 and 4.

To test how individual difference factors impacted confidence as a function of time, we regressed multiple two-way interactions between the different individual difference measures and median confidence RTs upon *z*-scored confidence as follows:

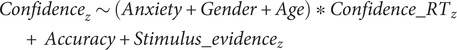

In the Supplementary Material, we also fitted the above two regression models additionally with transdiagnostic Compulsivity scores.

### Computational model

We extended recent work on drift-diffusion models (DDMs) of confidence that simultaneously account for decisions, RTs, and confidence on two-alternative forced-choice tasks (Desender et al., 2022; Hellmann et al., 2023; Pleskac & Busemeyer, 2010) to also model distortions in confidence formation during the post-decisional period. In these models, evidence *e* accumulates over time at a drift rate ( 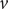 ) until it reaches a decision bound corresponding to one of the two alternatives (by convention, 0 and 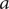 ) at which time a decision is made. Evidence accumulation begins at an initial bias value of 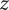 (< 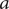 ), and after nondecision time, τ. For each time increment, 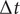 , accumulated evidence is then:

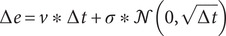

Here, 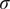 is the noise of the accumulation process, and is conventionally set to 1, as it trades off with 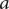 (Desender et al., 2022).

In post-decisional DDMs, evidence continues to accumulate following a decision, accounting for the improved correspondence between decision and confidence (Pleskac & Busemeyer, 2010). Recent work has shown, moreover, that post-decisional evidence may accumulate at a different drift rate ( 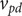 ) than the one used to make the initial decision. Here, the ratio 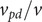 ( 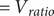 ) reflects a dynamic measure of metacognitive efficiency for an individual (Desender et al., 2022). Within such a model, we propose that the post-decisional drift-rate could additionally be biased by a factor 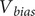 in favour of or against the decision. Thus, on an individual task trial, 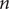 , where a participant made the decision 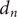 (= +1 or − 1 corresponding to upper and lower decision bounds, respectively), a biased post-decisional drift rate will be:

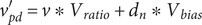

Following Desender et al. (2022), we sampled (with replacement) from empirical distributions of confidence response times ( 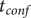 ) for each subject to estimate the accumulated post-decisional evidence. Using the formula of a Wiener process distribution for a given time interval and drift, the final evidence used for reporting confidence is then:

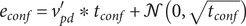

Finally, this confidence evidence on a continuous scale is transformed into a bounded probabilistic estimate of confidence. To achieve this transformation, we use a logistic function (although in principle this could also be achieved through other cumulative distribution functions of continuous probability distributions (Guggenmos, 2022). This process introduces two more potential sources of distortion, namely, additive ( 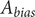 ) and multiplicative (or slope, 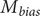 ) shifts of the criteria used to form confidence, as follows:

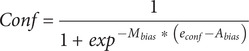

The model has eight free parameters ( 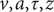 , 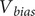 , 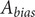 , 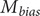 , and 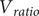 ) and fits the observed choices, choice RTs, and confidence reports. To fit the model, we followed a procedure similar to Desender et al. (2022) with one key difference. We split the observed choice, RTs, and confidence distributions into five quantiles (.1, .3, .5, .7, and .9). We then simulated the model for a large number of iterations (4,000) for combinations of the eight free parameters generated using differential evolution optimization implemented in R using the DEOptim package (Mullen et al., 2009). For optimization, we used the following chi-squared error function:

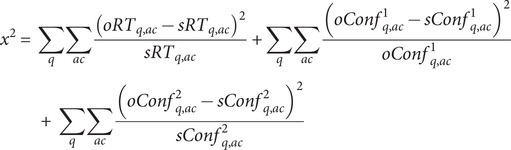

Here, 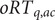 and 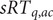 are the observed and simulated RTs for each quantile and accuracy (i.e. on correct and incorrect trials). To fit confidence reports, we split the data into two halves based on whether confidence ratings were faster or slower than the median confidence (i.e. post-decisional) RTs. This allowed us to simultaneously fit the three bias parameters because the model makes different predictions for confidence in relation to fast and slow confidence RTs (Figure 3d–e and Supplementary Figure 2). 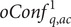 and 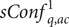 are observed and simulated confidence values for fast confidence RTs, while 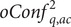 and 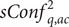 are confidence values for slow confidence RTs. For fitting, we used 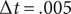 . For experiments where confidence reports were provided on a 5- or 11-point Likert scale (i.e. not on a continuous scale), we fit distributions for each confidence level instead of quantiles. In experiments with more than one task, the model was fit separately for each task, and *z*-scored parameters were averaged for each participant before statistical analyses.

To test the relationship between each individual difference factor and fitted model parameters (see below), we constructed regression models as follows:

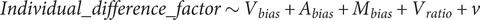

For these regression models, we excluded extreme outliers for V-ratio, V-bias, and A-bias that were > 5 standard deviations from the mean within each experiment and task (results were similar for more conservative or liberal exclusion criteria). In Supplementary Material, we report additional analyses addressing open questions about the domain generality of dynamic metacognitive efficiency and bias parameters, as well as population-level metacognitive biases.

## Results

We asked how two distinct individual difference factors – Anxiety and Gender – influenced confidence over time by pooling data from four large existing datasets obtained from two recent studies (Katyal et al., 2025; Rouault et al., 2018) with a combined *N* = 1,447 (Experiment 1: *N* = 300, Experiment 2: *N* = 387, Experiment 3: *N* = 433, Experiment 4: *N* = 327). Here, we report analyses from the combined dataset, noting any deviations between datasets.

### Underconfidence associated with gender and anxiety symptoms

We first replicated previous work showing the influence of Anxiety and Gender on reported confidence in the combined dataset. As expected, Anxiety scores were negatively associated with confidence level (Supplementary Figure 1A; 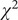 = 44.90, *p* = 2.3e-11). We also found that women exhibited lower confidence than men (Supplementary Figure 1B; 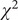 = 5.01, *p* = .025).

### Different dynamics of underconfidence in relation to gender and anxiety symptoms

**Figure 2.**
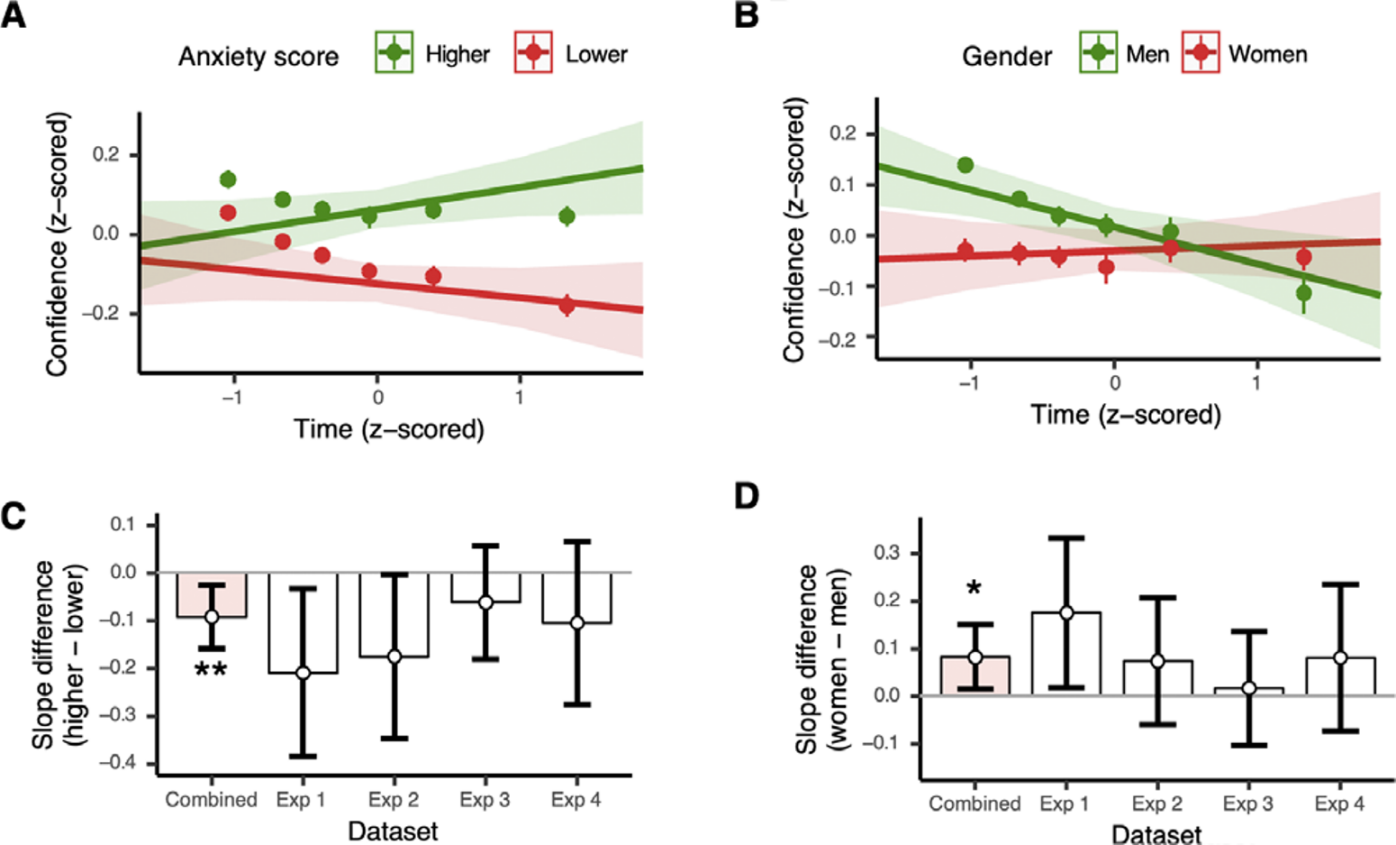
(a) Underconfidence in relation to Anxiety symptom scores increases with post-decision time (*z*-scored within each dataset and task). (b) Conversely, underconfidence in relation to Gender decreases with post-decision time. (a), (b) Lines show marginal effects of two-way interactions of individual difference factors with post-decision time regressed upon *z*-scored mean confidence. Dots show regression predicted confidence data tiled into six time bins. (c), (d) The difference in slopes obtained from the same regression models between higher and lower Anxiety scores (c) and women and men (d). (c) Increasing underconfidence in relation to Anxiety symptoms is observed in the combined dataset, as well as individually in Experiments 1 and 2. (d) Decreasing underconfidence in relation to Gender is observed in the combined dataset, as well as in Experiment 1. Error bars show 95% bootstrapped confidence intervals. **p < .01, *p < .05.

Next, we asked how distortions in confidence related to the two individual difference factors as a function of post-decision time. We found that the difference in confidence between lower and higher Anxiety scores became larger as individuals took longer to report their confidence, leading to an increase in underconfidence with time (red and green lines are diverging; Figure 2a). In contrast, the difference in confidence associated with Gender was greatest immediately following decisions and reduced as individuals took longer to report their confidence (red and green lines are converging; Figure 2b). We statistically evaluated these effects by computing two-way interactions between individual difference factors and mean post-decision time in association with reported confidence (see Methods). Here, an increase in underconfidence with time would be observed as a negative difference in slopes between high and low values of the individual difference measure. Conversely, a decrease in underconfidence with time would be observed as a positive difference in slopes between high and low values of the measure.

We found a significant interaction between Anxiety symptoms and post-decision time in association with reported confidence ( 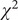 = 7.30, *p* = .007; Figure 2a), with a *negative* slope difference between high and low Anxiety scores (Figure 2c). The effect was also observed individually in Experiments 1 and 2, along with a trend in the same direction in Experiments 3 and 4. For Gender, there was also a significant interaction with post-decision time in association with reported confidence ( 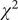 = 5.80, *p* = .016; Figure 2b), with a *positive* slope difference between women and men (Figure 2d). The effect was observed in Experiment 1 with a trend in the same direction in Experiments 2 and 4.

### Computational models of how individual differences affect confidence

Building on recent work (Desender et al., 2022; Pleskac & Busemeyer, 2010), we developed a computational model of how changes in post-decisional processing generate distorted confidence reports. Post-decisional accounts of confidence in two-choice tasks are grounded in classical drift-diffusion/evidence accumulation models of decision-making (Calder-Travis, Bogacz, & Yeung, 2023; Pleskac & Busemeyer, 2010; Yeung & Summerfield, 2012). In these models, evidence accumulates at a particular drift rate (corrupted by noise) until it reaches one of two bounds, corresponding to the two possible choices. This bound-crossing determines the time and accuracy of the first-order decision. Following the decision, evidence continues to accumulate with a post-decisional drift rate. Confidence in the decision is proportional to the total accumulated evidence at the time the agent provides the confidence report. The ratio of the pre- and post-decisional drift rate (known as “v-ratio”) reflects a dynamic measure of metacognitive efficiency (Desender et al., 2022). V-ratio is found to be typically >0, indicating an increase in metacognitive efficiency with post-decisional time, but also <1, indicating that the post-decisional drift rate is lower than the pre-decisional drift rate (Desender et al., 2022; Pleskac & Busemeyer, 2010).

**Figure 3.**
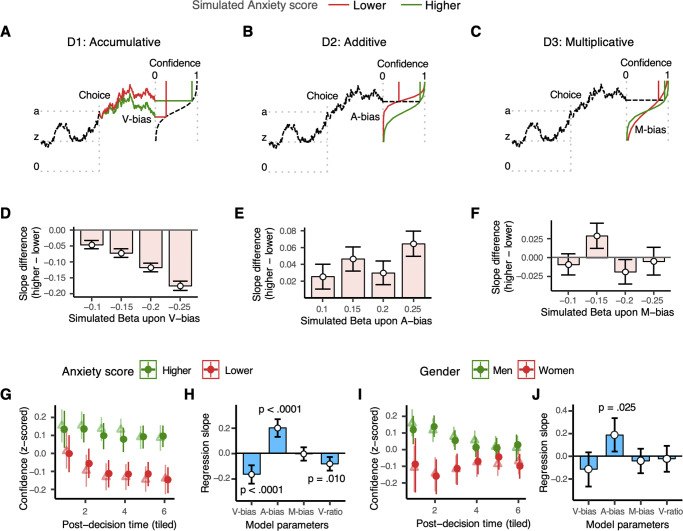
Three accounts of how confidence distortions could be generated for the paradigmatic case of underconfidence in relation to simulated Anxiety symptoms. (a) D1: Accumulative – distorted accumulation of evidence over time after making a decision. (b) D2: Additive – the criterion for reporting higher confidence is shifted to a higher level of evidence. (c) D3: Multiplicative – evidence is more gradually converted into extreme confidence values. (d)–(f) Difference in regression slopes relating post-decision time to confidence between higher and lower Anxiety scores (as in Figure 1) for a range of model parameters similar to fitted parameters from observed data (Supplementary Figure 2 shows similar plots with a larger range of simulated parameters). (d) Negative values show that for D1, distortion increases with time. (e) For D2, distortion decreases with time. (f) For D3, distortion can increase, decrease, or not change with time, depending on other parameters. Model fitted confidence (open triangles) depicted besides observed data from Experiments 1 and 2 combined (filled circles), split by (g) higher and lower Anxiety scores, and (i) women and men (similar plots for Experiments 2 and 3 shown in Supplementary Figure 4). The model can capture the opposite relationships between confidence distortion and time observed for individual differences in Anxiety symptoms and Gender. Regression slopes for four parameters recovered from the model, V-bias (drift rate/accumulation distortion), A-bias (additive distortion), M-bias (multiplicative distortion), and V-ratio (metacognitive efficiency), in relation to (h) Anxiety scores, and (j) Gender. For (g)–(h), error bars show 95% confidence intervals across participants.

We extended this account to model different sources of metacognitive distortion for the paradigmatic case of underconfidence in relation to Anxiety symptoms (Figure 3a–f). We simulated populations in which an individual’s confidence was distorted by Anxiety symptom scores according to three candidate mechanisms (see Methods for details): (D1) Accumulative distortion – the post-decisional drift rate is negatively biased in proportion to the Anxiety score (V-bias), resulting in lower evidence favoring the decision at the time of the confidence response and thus lower confidence (Figure 3a); (D2) Additive distortion – an additive shift in the confidence criterion proportional to the Anxiety score (A-bias) with higher criteria resulting in lower confidence and leading to a lateral shift in the sigmoid function that transforms (unbounded) evidence to bounded confidence reports (Figure 3b); (D3) Multiplicative distortion – the slope of the sigmoid function relating confidence to evidence is inversely related to Anxiety scores (M-bias), such that individuals with high Anxiety scores more gradually increase from lower to higher confidence as accumulated evidence increases (Figure 3c). Figure 3a–c depict how all three mechanisms can lead to underconfidence. Simulating the model at a population level with these three mechanisms across a range of model parameters confirmed that D1 and D2 always lead to underconfidence (Supplementary Figure 2A and 2C), whereas D3 leads to underconfidence for some combination of parameter values and overconfidence for others (Supplementary Figure 2E).

The three mechanisms also exhibited different patterns for how an individual difference factor impacts confidence as a function of time after the decision. Figure 3d–f plot the difference in regression slopes relating post-decision times to confidence between higher and lower values of (simulated) Anxiety scores for the three types of distortions (Supplementary Figure 2B, 2D, and 2F show similar plots for a larger range of parameter values). Simulations showed that confidence distortion (here, underconfidence) due to D1 generally increases with greater post-decision time – manifesting as a negative slope difference between higher and lower values of simulated Anxiety scores (Figure 3d and Supplementary Figure 2B). Mechanism D1 thus qualitatively matches patterns observed for underconfidence in relation to Anxiety symptoms (Figure 2a). Conversely, for D2, confidence distortion generally decreases with greater post-decision time – this is seen as a positive slope difference (Figure 2b). Mechanism D3, however, did not show a consistent pattern and could lead to an increase, decrease, or no change in confidence distortion over time, depending on the choice of other parameters.

Next, we fit the model to individual participant data to obtain four post-decisional confidence parameters that characterized each participant’s confidence distortions: V-bias, A-bias, M-bias, and V-ratio (a dynamic measure of metacognitive efficiency (Desender et al., 2022). All parameters displayed excellent recoverability and identifiability in simulation (Supplementary Figure 3). Figure 3g and i show the fit of the model to participants separated by high/low Anxiety scores and Gender, combined across Experiments 1 and 2 (similar plots for Experiments 3 and 4 can be found in Supplementary Figure 4). Importantly, the model can capture the opposite relationships between underconfidence and time observed for individual differences in Anxiety symptoms and Gender.

### Distinct computational sources of underconfidence associated with gender and anxiety symptoms

We next regressed fitted model parameters on the two individual difference measures (Figure 3h, j). Notably, Anxiety scores were significantly negatively associated with V-bias ( 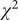 = 19.9, *p* = 3.5e–05), confirming our expectation that individuals with high Anxiety symptoms accumulate negatively biased evidence about their performance as time elapses after a decision. Higher Anxiety symptoms were also significantly positively associated with an additive shift in confidence criteria ( 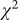 = 31.4, *p* = 1.8e-07; it is noteworthy that positive shifts in criteria shift correspond to underconfidence) but not by a multiplicative shift ( 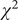 = .02, *p* = .90). Anxiety symptoms were also significantly negatively associated with V-ratio ( 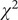 = 8.4, *p* = .010), indicating poorer (dynamic) metacognitive efficiency. In contrast, Gender was significantly positively associated with A-bias ( 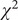 = 6.4, *p* = .025), but not by V-bias ( 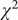 = 2.2, *p* = .23). Gender was also not associated with M-bias ( 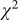 = .6, *p* = .61) or V-ratio ( 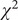 =.2, *p* = .80). Thus, gender-related underconfidence in women was associated with an additive shift in the confidence criterion rather than a negatively biased accumulation of evidence following a decision.

## Discussion

Confidence in abilities determines the kinds of tasks people pursue in everyday life (Bandura, 1977). Indeed, in the absence of external feedback, confidence provides a summary belief about one’s skill or ability (Katyal et al., 2025; Rouault, Dayan, & Fleming, 2019). However, a number of individual difference factors are known to distort people’s estimation of confidence in their abilities. Prominently, both anxiety (Benwell et al., 2022; Hoven et al., 2023; Rouault et al., 2018; Seow & Gillan, 2020), and gender (women compared to men; Cooke-Simpson & Voyer, 2007; Hoogervorst et al., 2024; Jakobsson, 2012; Lundeberg et al., 1994) are associated with underconfidence in one’s abilities, independently of objective performance. Here, we investigated and modeled how these confidence distortions unfold with time. Specifically, we asked whether underconfidence associated with anxiety symptoms is computationally similar or distinct from underconfidence related to gender.

To investigate these questions, we pooled data from four large online datasets (Katyal, Huys, Dolan, & Fleming, 2025; Rouault et al., 2018). Our key observation was that anxiety symptoms and gender led to opposite relationships between post-decision time and confidence. Underconfidence associated with anxiety increased with post-decision time, whereas underconfidence in women decreased with post-decision time. At a psychological level, one interpretation of these findings is that providing more time for post-decisional introspection or reflection can either deepen or ameliorate underconfidence, depending on the source of the bias. In the case of anxiety, it is plausible that greater time for post-decisional reflection leads to an increase in negative thoughts about one’s performance, similar to the effects of brief bouts of rumination (Watkins & Roberts, 2020). In contrast, the effect observed in relation to gender suggests that gender-related underconfidence is a prepotent bias – perhaps stemming from social factors – and which can be overcome by additional reflection on one’s actual performance. Indeed, at the longest post-decision response times, there was no longer any difference in confidence between genders – offering a potential explanation for the heterogeneity in previous work relating gender and confidence (Hoogervorst et al., 2024; Xue et al., 2024).

To account for these differences, we developed a computational model in which confidence ratings are generated with three distinct sources of metacognitive bias in addition to dynamic metacognitive efficiency (Desender et al., 2022). Model fits revealed that while both anxiety symptoms and gender were associated with an additive shift in confidence criteria, only anxiety symptoms led to a negatively biased accumulation of evidence about performance over time. This is consistent with anxiety symptoms triggering negatively biased reflections on performance and exacerbating underconfidence with additional time for introspection. In contrast, underconfidence in relation to gender was explained by an initial shift in confidence criteria that can be overcome with additional evidence, thus leading to improved introspective accuracy and a reduction in underconfidence with increased time for introspection (Desender et al., 2022; Pleskac & Busemeyer, 2010).

Our results thus suggest straightforward prescriptions for reducing confidence distortions in relation to different classes of individual differences. For individuals with anxiety symptoms, it may be better to rely more on early confidence estimates formed immediately after completing a task, rather than letting negative biases creep in with additional time for introspection. On the contrary, women may benefit from taking additional time to evaluate their abilities, allowing a boost in confidence to accrue from the accumulation of (unbiased) evidence about their performance and reducing the influence of preexisting negative biases. We note that underconfidence in women here is characterized in relative, rather than absolute, terms – and is expressed relative to the confidence level held by men. In other words, these recommendations also hold for ameliorating a relative overconfidence in men, which should be similarly reduced by taking additional time to evaluate one’s performance.

Our novel computational model of dynamic metacognition not only models the dynamics of metacognitive efficiency (Desender et al., 2022, but also three distinct sources of metacognitive bias. Several studies reveal distinct factors that impact metacognitive bias without changing underlying performance, including affect (Allen et al., 2016), reward expectation (Lebreton et al., 2018; Salem-Garcia, Palminteri, & Lebreton, 2023), priors on performance (Van Marcke et al., 2024), and feedback (Katyal et al., 2025). Future work can utilize our model to provide computational insights into how these different factors distort confidence over time.

Besides gender and anxiety, we also explored the parameters of our model in relation to individual differences in age and transdiagnostic compulsivity (see Supplementary Material). We found an increase in underconfidence over time for older versus younger individuals, which was explained by a reduced multiplicative bias. We also found an increase in overconfidence over time with compulsivity, which was explained by a positively biased accumulation of evidence and additive response bias. These additional investigations suggest that distortions in post-decisional processing may constitute a general mechanism underlying different manifestations of metacognitive distortions in relation to mental health.

Although our main analyses involved pooling data from four existing large online experiments, we also analyzed each experiment individually. Our findings of how confidence distortions unfolded over time in relation to gender and anxiety were largely consistent in all but one (Experiment 3) experiment. We suspect this difference may be due to methodological features of Experiment 3. While other experiments included a psychophysical staircase to adaptively control task difficulty, Experiment 3 used a method of constant stimuli in which fixed levels of task difficulty are randomly varied across trials. It is plausible that the influence of task difficulty on confidence may have masked the presence of confidence distortions. For example, easy and hard trials may exhibit ceiling and floor levels of confidence and be less impacted by biases. In contrast, the use of an adaptive staircase may ensure an intermediate level of uncertainty in participants about their decision, where distortions due to individual difference factors become exposed.

In summary, we found that underconfidence associated with anxiety symptoms became more prominent the longer individuals took to make metacognitive judgments – suggesting that an underconfidence related to mental ill health is exacerbated by additional introspection. In contrast, gender-related underconfidence decreased over time – suggesting that taking more time to consider one’s performance remediates prepotent biases. A novel dynamic computational model of confidence explained these effects – while both gender and anxiety symptoms involved shifts in confidence criteria, only anxiety symptoms were linked to the accumulation of negatively biased evidence about one’s ability. Our study reveals distinct computational sources of underconfidence, in turn highlighting potential mechanisms for its remediation.

## Supporting information

10.1017/S0033291725102808.sm001 Katyal and Fleming supplementary material Katyal and Fleming supplementary material

## Data Availability

Data are publicly available at github.com/sucharitk/underconfidence/. All code for data analysis and figure generation is publicly available at github.com/sucharitk/underconfidence/.

## Supplementary material

The supplementary material for this article can be found at http://doi.org/10.1017/S0033291725102808.
