# Supplementary material for: Gender and anxiety reveal distinct computational sources of underconfidence
Source: Psychol Med. 2026 Jan 15;56:e17. doi: 10.1017/S0033291725102808 (PMC12885344; doi:10.1017/S0033291725102808)
Supplement: Katyal and Fleming supplementary material [file S0033291725102808sup001.docx]

**Supplementary Material**

**Gender and anxiety reveal distinct computational sources of underconfidence**

Sucharit Katyal^1,4^, Stephen M Fleming^1,2,3^

^1^ Max Planck UCL Centre for Computational Psychiatry and Ageing Research, University College London

^2^ Wellcome Centre for Human Neuroimaging, Queen Square Institute of Neurology, University College London

^3^ Department of Experimental Psychology, University College London

^4^ Department of Psychology, University of Copenhagen

**Supplementary Tables**

Supplementary Table 1. Characteristics of the four datasets used in our study including which individual difference measures were and were not available in each dataset

| Exp | Original dataset | N | N (after exclusions) | Anxiety | Compulsivity | Age and Gender | Tasks |
| --- | --- | --- | --- | --- | --- | --- | --- |
| 1 | Katyal et al, 2023, Exp 1 | 300 | 300 | ✅ | ❌ | ✅ | 1: Perception  2: Working memory |
| 2 | Katyal et al, 2023, Exp 2 | 387 | 387 | ✅ | ✅ | ✅ | 1: Perception  2: Working memory |
| 3 | Rouault et al, 2018, Exp 1 | 483* | 433 | ✅ | ❌ | ✅ | Perception |
| 4 | Rouault et al, 2018, Exp 2 | 477* | 327 | ✅ | ✅ | ✅ | Perception |

*The original study Rouault et al (2018) reported N = 498 and N = 497 for Exp 3 and 4 respectively. However, individual trial confidence response time data was only available for the Ns reported here.

**Supplementary Results**

**Confidence distortions by Age and Compulsivity**

We also evaluated how post-decision time impacted confidence distortions in relation to transdiagnostic symptoms of Compulsivity—which have been associated with overconfidence (Benwell et al., 2022; Rouault et al., 2018; Seow & Gillan, 2020)—and Age—which has been associated with underconfidence in some studies (McWilliams, Bibby, Steinbeis, David, & Fleming, 2023) but overconfidence or an absence of confidence distortion in others (Meunier et al., 2024; Scarampi & Gilbert, 2021).

*Age*

In our combined dataset, we found that Age negatively predicted confidence level ($\chi^{2}$ = 23.00, p = 1.7e-06; Supplementary Figure 1C). There was however no change in age-related underconfidence with post-decision time with no significant 2-way interaction between Age and post-decision time regressed upon confidence ($\chi^{2}$ = .04, p = .85).

When fitting the model to data, we observed that Age was strongly negatively associated with M-bias ($\chi^{2}$ = 25.5, p = 4.5e-07; Supplementary Figure 5A). In other words, older individuals tended to use a larger range of the rating scale compared to younger individuals. Age was also associated with A-bias ($\chi^{2}$ = 3.9, p = .048) but not V-bias ($\chi^{2}$ = .03, p = .86) or V-ratio ($\chi^{2}$ = .7, p = .40).

*Compulsivity*

Measures of transdiagnostic Compulsivity were only available for two of the four experiments – Exp 2 and Exp 4 (combined N = 714). As expected, Compulsivity scores positively predicted confidence level after controlling for Anxiety scores (Supplementary Figure 1D; $\chi^{2}$ = 18.25, p = 2.1e-05), consistent with several previous studies (Benwell et al., 2022; Katyal et al., 2023; Rouault et al., 2018; Seow & Gillan, 2020). However, there was no significant interaction between Compulsivity and post-decision time when predicting reported confidence ($\chi^{2}$ = 1.00, p = .32).

Model fitting revealed that Compulsivity scores were significantly negatively associated with A-bias ($\chi^{2}$ = 11.1, p = .0009; Supplementary Figure 5B) and V-bias ($\chi^{2}$ = 4.6, p = .032) but not M-bias ($\chi^{2}$ = 1.6, p = .21) or V-ratio ($\chi^{2}$ = .03, p = .87). Thus, similar to the pattern observed for Anxiety symptoms, higher Compulsivity symptoms were related to distorted accumulation of evidence as time elapsed following a decision — in this case, an accumulation of positively-biased evidence. Additionally, Compulsivity symptoms were related to a negative shift of the additive confidence criterion.

When considered together with the pattern observed for Anxiety symptoms, it is notable that confidence distortions related to psychiatric symptoms (Hoven et al., 2019) tended to accumulate over time indicating distortions in post-decisional sampling. On the other hand, confidence distortions induced by non-psychiatric factors—like Gender and Age—involve prepotent biases that manifest in shifts in metacognitive criteria.

**Dynamic metacognitive efficiency and domain specificity/generality**

Static measures of metacognitive efficiency (e.g., M-ratio) have consistently revealed better metacognition for memory than perception (Lund, Correa, Fardo, Fleming, & Allen, 2023; Mazancieux, Fleming, Souchay, & Moulin, 2020; Morales, Lau, & Fleming, 2018). However, this difference could be due to domain-specific differences in post-decisional time that contribute to dynamic measures of metacognitive efficiency (Desender et al., 2022). Similarly, Desender (2022) suggest that some aspects of domain-generality in in metacognition (such as the correlation between metacognition for perception and memory; (Lund et al., 2023; Mazancieux et al., 2020; McCurdy et al., 2013)) could potentially be explained by generalised individual differences in response caution. Because Exp 1 and Exp 2 used both perception and memory tasks, we are able to to ask 1) if dynamic metacognitive efficiency is greater for memory compared to perception, and 2) if dynamic metacognitive efficiency is domain-general, i.e., individuals with higher V-ratio for perception also have higher V-ratio for memory, controlling for individual differences in post-decision time.

We first tested if participants had good metacognition, on average, using the dynamic measure of metacognitive efficiency, V-ratio. This would be observed as V-ratios > 0. Consistent with recent work (Desender et al., 2022), in all experiments, we observed V-ratios > 0.

Supplementary Table 2. For each experiment and task, characteristics of the V-ratio parameter including the statistical comparison V-ratio > 0

| Experiment | Task | Mean  V-ratio | 95% Confidence Interval | Student’s t and degrees of freedom | p-value |
| --- | --- | --- | --- | --- | --- |
| 1 | Perception | .262 | [.223 .298] | 14.36 (227) | < 2.2e-16 |
|  | Memory | .588 | [.525 .651] | 18.56 (229) | < 2.2e-16 |
| 2 | Perception | .250 | [.218 .283] | 15.14 (379) | < 2.2e-16 |
|  | Memory | .722 | [.659 .785] | 22.46 (375) | < 2.2e-16 |
| 3 | Perception | .298 | [.282 .314] | 36.13 (476) | < 2.2e-16 |
| 4 | Perception | .279 | [.265 .292] | 40.61 (473) | < 2.2e-16 |

Interestingly, even though perception tasks were substantially different between the two sets of studies (i.e., between Experiments 1 & 2 versus Experiments 3 & 4), their V-ratios were similar (averaging between .2 and .3).

Next, we compared metacognitive efficiency between the two tasks. In Exp 1, half the participants performed both perception and memory tasks while the other half performed only one of the two tasks. For a conservative statistical estimate (i.e., ignoring within-subject variance), we performed a two-sample Welch t-test and found V-ratios were significantly larger for memory compared to perception (t(365.2) = 8.93; p < 2.2e-16; 95% CI = [.254 .398]). In Exp 2, all participants performed both tasks allowing use of a paired t-test. We again found V-ratio to be significantly larger for memory compared to perception (t(369) = 13.74; p < 2.2e-16; 95% CI = [.41 .55]). There was thus strong evidence for better metacognitive capacity for memory compared to the perception tasks, even when controlling for differences in post-decision time.

To test for domain-generality, we performed Pearson correlations of V-ratio between the perception and memory tasks. In both Exp 1 (r = .139; t(153) = 1.73; p = .085) and Exp 2 (r = .010; t(368) = 1.91; p = .057), we found a trend for positive correlations, consistent with some degree of domain-generality in dynamic metacognitive efficiency. To quantify the overall evidence for domain-generality from the two experiments, we conducted a Bayes Factor analysis by combining the two samples and using default priors from the *BayesFactor* package in R. This revealed decisive evidence in favour of a correlation between V-ratios for perception and memory (BF = 4.28e+50 ± 0%). Together, these analyses suggest that domain-generality in metacognitive efficiency survives control for variation in post-decisional time, suggesting that a generalised response caution is unable to fully account for the effect.

**Overconfidence at a population level**

At a population level, overconfidence is commonly found on a variety of tasks (Klayman, Soll, González-Vallejo, & Barlas, 1999; West & Stanovich, 1997). However, the extent of overconfidence has rarely been assessed using model-based metrics that account both for both first- and second-order abilities (although see Mamassian & de Gardelle, 2021), or within a dynamic model of metacognition. We tested if our two key model-based metrics of confidence bias, namely shifts in the response criterion (A-bias) and accumulative bias (V-bias), showed population-level characteristics of overconfidence.

For A-bias, overconfidence would be observed as a significantly negative value of A-bias in the population (lower criterion corresponds to higher confidence bias). Supplementary Table 3 shows that in all four experiments and within both tasks for Exp 1 and 2, there was a negative shift in response criterion reflecting a general bias towards overconfidence. Previous work suggested that the degree of overconfidence may differ between tasks (Klayman et al., 1999). We thus tested if overconfidence was larger in one task than the other. In both Exp 1 (Welch t-test; t(449.08) = 3.14; p = .0018; 95% CI = [.241 1.051]) and Exp 2 (paired t-test; t(372) = 5.33; p = 1.7e-7; 95% CI = [.496 1.076]), we found that overconfidence was significantly larger in the perception than memory tasks. The A-bias parameter also exhibited domain-generality such that individuals who were more overconfident in the perception task were also more overconfident in the memory task (Exp 1: r = .483; t(153) = 6.82; p = 2.0e-10; Exp 2: r = .227; t(371) = 4.50; p = 9.3e-06).

Supplementary Table 3. For each experiment and task, characteristics of the A-bias parameter including the statistical comparison A-bias > 0

| Experiment | Task | Mean | 95% Confidence Interval | Student’s t (degrees of freedom) | p-value |
| --- | --- | --- | --- | --- | --- |
| 1 | Perception | –.955 | [–1.260 –.650] | –6.17 (228) | < 3.18e-09 |
|  | Memory | –.309 | [–.576 –.041] | –2.27 (229) | .024 |
| 2 | Perception | –1.003 | [–1.236 –.770] | –8.47 (382) | 5.52e-16 |
|  | Memory | –.236 | [–.470 –.004] | –2.00 (375) | .047 |
| 3 | Perception | –1.311 | [–1.469 –1.153] | –16.33 (479) | < 2.2e-16 |
| 4 | Perception | –.336 | [–.539 –.133] | –3.26 (474) | .0012 |

We also tested if the V-bias parameter exhibited a positively-biased accumulation of evidence about performance at the population level (Supplementary Table 4), with the expectation that average V-bias > 0. However, while V-bias > 0 was observed in the memory tasks in both Exp 1 & 2, results were mixed with regards to the perception tasks in Exp 1–4. We quantified evidence for overconfidence separately in the perception and memory tasks by calculating Bayes Factors. For the memory task combined for Exp 1 and 2 we found decisive evidence for a population-level overconfidence in V-bias (BF = 166.80 ± 0%). However, for perception tasks (combined for Exp 1–4), we found strong evidence in favour of the null hypothesis indicating the lack of population-level overconfidence effect (BF = .048 ± .48%).

Supplementary Table 4. For each experiment and task, characteristics of the V-bias parameter including the statistical comparison V-bias > 0

| Experiment | Task | Mean | 95% Confidence Interval | Student’s t (degrees of freedom) | p-value |
| --- | --- | --- | --- | --- | --- |
| 1 | Perception | –.016 | [–.198 .166] | –.18 (228) | .86 |
|  | Memory | .242 | [.096 .388] | 3.26 (229) | .001 |
| 2 | Perception | –.182 | [–.324 –.039] | –2.50 (382) | .013 |
|  | Memory | .206 | [.061 .351] | 2.79 (375) | .006 |
| 3 | Perception | .079 | [.005 .153] | 2.09 (477) | .038 |
| 4 | Perception | –.019 | [–.095 .057] | –.49 (473) | .63 |

**Supplementary Figures**

**Supplementary Figure 1.** Regression slopes of four inter-individual factors upon confidence for the combined (yellow bars) and individual (white) datasets, **A)** Anxious-Depression, **B)** Gender, **C)** Age, and **D)** Compulsivity. Error bars show SEMs. ****p < .0001, ***p < .001, **p < .01, *p < .05.

**Supplementary Figure 2.** Left panels: Regression slope of a simulated individual factor AD (Anxious-Depression) upon model-generated confidence where the model was simulated with the three types of distortions **A)** accumulative, **C)** additive**,** and **E)** multiplicative, for a range of model parameters. X-axis shows different simulated regression slopes upon the distortions. M0 and A0 are different intercept values for multiplicative and additive biases respectively. **A–C)** show that all distortions can exhibit underconfidence. Right panels: Slope difference of high vs. low simulated AD scores for the three distortions **B)** accumulative, **D)** additive**,** and **F)** multiplicative.

**Supplementary Figure 3.** Parameter recovery for the four key parameters of the computational model **A)** V-bias, **B)** A-bias, **C)** M-bias, and **D)** V-ratio. **E)** Correlation matrix showing all combinations of the four simulated and recovered parameters showing that the model does not induce artificial correlations between parameters.

**Supplementary Figure 4.** Model fits (open triangles) depicted besides observed data from Exp 3 and Exp 4 combined (filled circles) averaged over correct and incorrect trials for **A)** higher and lower Anxious-Depression scores, and **G)** women and men.

**Supplementary Figure 5.** Regression slopes for the four key model parameters, V-bias (drift-rate/accumulation distortion), A-bias (additive distortion), M-bias (multiplicative distortion) and V-ratio (metacognitive efficiency), upon **A)** Age and **B)** Compulsivity scores. Error bars show 95% confidence intervals across participants. P-values not corrected for multiple comparisons.
